# PNPLA3 and TLL-1 Polymorphisms as Potential Predictors of Disease Severity in Patients With COVID-19

**DOI:** 10.3389/fcell.2021.627914

**Published:** 2021-06-23

**Authors:** Stefania Grimaudo, Emanuele Amodio, Rosaria Maria Pipitone, Carmelo Massimo Maida, Stefano Pizzo, Tullio Prestileo, Fabio Tramuto, Davide Sardina, Francesco Vitale, Alessandra Casuccio, Antonio Craxì

**Affiliations:** ^1^Department of Health Promotion, Mother and Child Care, Internal Medicine and Medical Specialties “G. D’Alessandro” – University of Palermo, Palermo, Italy; ^2^Infectious Diseases Unit and Centre for Migration and Health ARNAS, Ospedale Civico Benfratelli, Palermo, Italy

**Keywords:** COVID-19, genetic polymorphism, severity of disease, TLL-1, PNPLA3 I148M

## Abstract

Albeit the pathogenesis of COVID-19 remains unclear, host’s genetic polymorphisms in genes involved in infection and reinfection, inflammation, or immune stimulation could play a role in determining the course and outcome. We studied in the early phase of pandemic consecutive patients (*N* = 383) with SARS-CoV-2 infection, whose subsequent clinical course was classified as mild or severe, the latter being characterized by admission to intensive therapy unit or death. Five host gene polymorphisms (MERTK rs4374383, PNPLA3 rs738409, TLL-1 rs17047200, IFNL3 rs1297860, and INFL4 rs368234815) were assessed by using whole nucleic acids extracted from nasopharyngeal swabs. Specific protease cleavage sites of TLL-1 on the SARS-CoV-2 Spike protein were predicted *in silico*. Male subjects and older patients were significantly at higher risk for a severe outcome (*p* = 0.02 and *p* < 0.001, respectively). By considering patients ≤65 years, after adjusting for potential confounding due to sex, an increased risk of severe outcome was found in subjects with the GG genotype of PNPLA3 (adj-OR: 4.69; 95% CI = 1.01–22.04) or TT genotype of TLL-1 (adj-OR=9.1; 95% CI = 1.45–57.3). *In silico* evaluation showed that TLL-1 is potentially involved in the Spike protein cleavage which is essential for viral binding and entry into the host cells using the host receptor angiotensin-converting enzyme 2 (ACE2). Subjects carrying a GG genotype in PNPLA3 gene might have a constitutive upregulation of the NLRP3 inflammasome and be more prone to tissue damage when infected by SARS-CoV-2. The TT genotype in TLL-1 gene might affect its protease activity on the SARS-CoV-2 Spike protein, enhancing the ability to infect or re-infect host’s cells. The untoward effect of these variants on disease course is evident in younger patients due to the relative absence of comorbidities as determinants of prognosis. In the unresolved pathogenetic scenery of COVID-19, the identification of genetic variants associates with more prolonged course or with a severe outcome of infection would support the development of predictive tools useful to stratify subjects by risk class at presentation. Moreover, the individuation of key genes could contribute to a better understanding of the pathways involved in the pathogenesis, giving the basis for rational therapeutic approaches.

## Introduction

More than 140 million cases of infection with Severe acute respiratory syndrome coronavirus 2 (SARS-CoV-2) have been reported worldwide by April 19, 2021, with over 3 million deaths due to the virus (World Health Organization, 2020 coronavirus disease situation dashboard. Available at^1^). While the clinical spectrum is extremely broad, ranging from mild or asymptomatic cases to severe acute respiratory syndrome, it has become immediately apparent that the outcome of infection is strongly conditioned by host-related factors such as age, gender and preexistent underlying illnesses. In the setting of RNA virus with a relatively fast evolutionary rate (Li et al., 2020) no clear evidence has instead emerged up to now for viral variability as a major determinant of pathogenicity. SARS-CoV-2 viral load, although linked to the phase of infection, does not appear to be a major determinant of pathogenicity even if a precise quantification of viral load in samples from oropharyngeal swabs remains elusive (Joynt and Wu, 2020).

Albeit the pathogenetic mechanism of coronavirus disease 2019 (COVID-19) is yet partly unclear, the course and outcome of disease seem to be significantly influenced by host factors. Although the role of virally driven hyperinflammation inducing a massive release of cytokines leading to a “Cytokine storm” is controversial (Sinha et al., 2020), the role of inflammatory processes in COVID-19 severity, especially in patients with comorbidities, is generally accepted (De la Rica et al., 2020). Since family clustering of severe cases was reported starting from the first phase of the pandemic, supporting the possibility of a genetic predisposition (Chan et al., 2020), it is thus conceivable that the complexity of host genetic background in terms of polymorphisms, may play a key role in pathogenesis and outcome of COVID-19. In this perspective, the COVID-19 Host Genetics Initiative, 2020 (available at COVID-19 HGI^2^) is currently leading a public effort worldwide to analyze COVID-19 information for millions of individuals in relation with genotype data to identify genetic variants associated with SARS-CoV-2 infection as well as COVID-19 hospitalization and disease severity (COVID-19 Host Genetics Initiative, 2020).

Several data arise from Genome-Wide Association study (GWAS) which, moreover, are not far from criticisms leading sometimes to the identification of variants that have not direct biological relevance to disease pathogenesis and being, on the other hand, not always able to discover all the variants involved in disease occurrence and progression (Tam et al., 2019). In this perspective together to GWAS, studies of candidate genes and cohort studies are still useful nowadays and this paper aims to evaluate Single Nucleotide Polymorphisms (SNP) in genes, whose products are known to interact with SARS-CoV-2 receptor-dependent endocytosis or involved in antiviral responses and inflammation conditioning host response to the virus.

We aimed to assess some specific functional SNPs of genes involved in control of viral infection by induction of inflammation (IFNL3/IFNL4), in macrophage polarization (MERTK), in tissue and systemic inflammation (PNPLA3). In addition, we evaluated a SNP of Tolloid Like-1 (TLL-1), a secreted protease capable of activating complement through the C1q pathway and also potentially able to activate the Spike protein of SARS-CoV-2. These SNPs were investigated on DNA samples directly derived from oropharyngeal swabs collected from a cohort of SARS-CoV-2 patients, from Sicily, Southern Italy, at the onset of the pandemic phase.

## Materials and Methods

Our observational study was carried out from February 24, 2020 to April 8, 2020 and included all consecutive Sicilian patients (*N* = 383) with laboratory-confirmed SARS-CoV-2 infection, whose oropharyngeal or nasopharyngeal swabs had been sent to the referral Laboratory for COVID-19 Surveillance for Western Sicily located at University Hospital “P. Giaccone” of Palermo. An approval to conduct the study has been required and obtained from the Ethical Committee of the A.O.U.P. “P. Giaccone” of Palermo, Italy (AIFA CE 150109 n. 03/2021).

Laboratory confirmation for SARS-CoV-2 was defined as a positive result of reverse transcriptase real-time polymerase chain reaction (rtReal-Time PCR) of nasal, pharyngeal or nasopharyngeal swabs according to the Centers for Disease Control and Prevention, 2020 protocol^3^. For each patient, sociodemographic variables (age, sex, and residency) were collected at the baseline, whereas clinical outcomes [home isolation, hospitalization, admission to intensive care unit (ICU), and death] were obtained by consulting the clinical profiles centrally provided by the Italian National Institute of Health (Istituto Superiore di Sanità, ISS) and, when available, by direct contact with the Hospitals involved in the care of each recruited patient. Each patient was monitored for at least 21 days after recruitment and the final day of follow-up was April 8, 2020. Due to the observational design, linked to referral, no follow up biological samples were available. Relatives were excluded from the cohort *a priori.*

### SNP Genotyping

Single Nucleotide Polymorphisms genotyping was carried out in 383 patients on the extracted whole nucleic acids from nasal or pharyngeal swabs (QIAamp Viral RNA Mini Kit, QIAGEN) by TaqMan genotyping allelic discrimination method (StepOne Plus Real Time PCR System, A.B. Foster City, CA, United States) using commercial (MERTK rs4374383, PNPLA3 rs738409, TLL-1 rs17047200) or custom (IFNL3 rs1297860, INFL4 rs368234815) genotyping assays (Thermo Fisher Scientific). Complete genotyping was not possible for all patients due to the scarce amount DNA available from swabs. The genotyping call was done by 2.3 Applied Biosystems Software. Genotyping was conducted in a blinded fashion relative to patient characteristics. Before testing for SNPs, samples were anonymized, and a unique randomly generated identification code was assigned to each record and to the correspondent swab. Researchers performing genetic analyses were unable to identify patients at all stages, and no permanent record linking these data to patient IDs was produced.

### Statistical Analysis

Statistical analyses were carried out by researchers not involved in the dataset storage and management. Calculation of the sample size was not performed *a priori*, the ultimate size being equal to the number of patients recruited during the entire study period. Continuous variables are presented as median and interquartile range (IQR) and categorical variables are expressed as number of patients (percentage).

For the purpose of most analyses, especially in relation to SNPs, patients were categorized into two main groups: mild disease (including those left isolated at home isolation and those hospitalized without complications) and severe disease (patients hospitalized for intensive/critical care and patients who died during the observation period, regardless of initial allocation).

Due to the presence of some missing data, the distribution of data over the age subgroups is based on the data available for each variable, while the remaining percentages are calculated using the number of data available for that subgroup. Univariate analysis was employed to identify variables associated with development of severe disease. Mann-Whitney rank sum test or ANOVA test were used to compare non-parametric continuous variables between age subgroups and patients with or without severe disease. Chi-square or Fisher exact tests were used for categorical variables as appropriate.

Multivariable logistic models were built to determine the association between potential confounders (age and sex) and the investigated genotypes. Each multivariable model included all patients in a first phase and only patients aged 65 years or less in a second phase. Due to the low frequency of some host’s genotypes, the multivariable models included only one genotype at time. All statistical tests were two-tailed, and statistical significance was defined as *P* ≤ 0.05. The analyses have not been adjusted for interaction, and given the possibility of type II error, the findings should be interpreted as exploratory and descriptive. Analyses were performed using (R Core Team, 2019) R Software analysis 3.6.1 (2019-07-05) (Available at^4^).

### Bioinformatic Analysis

For TLL-1, prediction of specific protease cleavage site on the target substrate was performed by SitePrediction (Verspurten et al., 2009). Top 20 predictions ranked by average score are reported.

Aminoacidic sequence in FASTA format of the target substrate Spike protein was retrieved from UniProt (accession: P0DTC2) that contained cleavage site specificity for members of the M12.016 sub-family (Rawlings et al., 2018).

## Results

Main features of 383 COVID-19 patients included in the study are summarized in Table 1. Overall, patient M/F ratio was 1.18 and median age was 58 years (IQR = 44–74 years), with a high percentage of subjects aged 41 to 64 years (39.95%). A total of 148 (38.64%) patients were hospitalized and 32 (8.36%) died during the follow-up period. Overall, 330 patients were classified as mild disease and 53 as severe disease.

**TABLE 1:** General features of the patient cohort (*N* = 383).

Sex, N (%)			
	- *F*	176	(45.95)
	- *M*	207	(54.05)
Age, N (%)			
	- 0–18	12	(3.13)
	- 19–40	66	(17.23)
	- 41–64	153	(39.95)
	- 65–74	60	(15.67)
	- >74	92	(24.02)
Age, median (IQR)			
		58	(44–74)
Hospitalization, N (%)			
	- *Yes*	148	(38.64)
	- *No*	235	(61.36)
Clinical outcome, N (%)			
	- *Home isolation*	234	(61.10)
	- *Hospitalization*	96	(25.06)
	- *Intensive/Critical care hospitalization*	21	(5.48)
	- *Death*	32	(8.36)

The distribution of gene polymorphisms in the entire population is reported in Table 2. One of the five investigated polymorphisms had an uncommon genotype (TT in TLL-1 rs17047200; *N* = 11, 2.96%), whereas low frequency genotypes were found in IFNL3 rs12979860 (TT *N* = 41, 10.7%), INFL4 rs368234815 (DG/DG 34, 11.30%), MERTK rs4374383 (AA in 44, 15.12%), and PNPLA3 rs738409 (GG 29, 7.82%), respectively. No significant difference was found between the expected frequency of these genotypes in the general Caucasian population (Phan et al., 2020) and that observed in our COVID-19 cohort.

**TABLE 2:** Distribution of host’s gene polymorphisms in the study cohort.

		*N*	%	Expected frequency in the Caucasian General population (%)
IFNL3 rs12979860 C > T (*N* = 383)	- CC	159	41.51%	47.61%
	- TC	183	47.78%	42.78%
	- TT	41	10.70%	9.61%
MERTK rs4374383 G > A* (*N* = 291)	- AA	44	15.12%	15.21%
	- GA	142	48.80%	47.58%
	- GG	105	36.08%	37.21%
INFL4 rs368234815 TT/DG* (*N* = 301)	- DG/DG	34	11.30%	9.61%
	- TT/DG	144	47.84%	42.78%
	- TT/TT	123	40.86%	47.61%
PNPLA3 rs738409 G > C* (*N* = 298)	- CC	199	54.64%	59.29%
	- CG	143	38.54%	35.42%
	- GG	29	7.82%	5.29%
TLL1 rs17047200 A > T*(*N* = 371)	- AA	285	76.82%	75.69%
	- AT	75	20.22%	22.62%
	- TT	11	2.96%	1.69%

*Not assessed in all subjects.

In the whole COVID-19 cohort the distribution of the genotypes of IFNL3, IFNL4, MERTK and PNPLA was in accord to the Hardy-Weinberg equilibrium, while the allelic distribution of the TLL-1 variant rs17047200 (A > T) (Table 3) showed a statistically significant divergence from Hardy Weinberg since the number of TT homozygotes observed was higher (11) than expected (6.3).

**TABLE 3:** Genotype distribution for TLL-1 rs10747200 in respect to Hardy-Weinberg equilibrium.

Genotypes	Observed	Expected
Homozygote AA	285	280.3
Heterozygote	75	84.3
Homozygote TT	11	6.3

*Variant Allele Frequency: 0.13; *X*^2^ = 4.532; X^2^ Test *p* value = 0.0332.*

Assessment of risk factors associated to a severe outcome is reported in Table 4. Male subjects and older patients were significantly at higher risk for a severe outcome (*p* = 0.02 and *p* < 0.001, respectively). In the entire cohort, none of the host’s SNPs was associated with COVID-19 severity of disease. When considering only patients aged 65 years or less, two genotypes were found to be significantly associated to an increased risk of severe outcome: GG for PNPLA3 rs738409 (*p* = 0.035) and TT for TLL-1 rs17047200 (*p* = 0.029), respectively.

**TABLE 4:** Risk factors associated with severe outcome (death or intensive/critical care hospitalization).

		Deceased/intensive care	Other conditions	*p*-Value
Sex, N (%)*	-*F*	16 (9.1)	160 (90.9)	0.02
	-*M*	37 (17.9)	170 (82.1)	
Age, median (IQR)		75 (67–79)	55 (42–71)	<0.001
IFNL3 rs12979860 C > T, N (%)				
	CC	24 (15.1)	135 (84.9)	
	TC	24 (13.1)	159 (86.9)	0.82
	TT	5 (12.2)	36 (87.8)	
MERTK rs4374383 G > A, N (%)*				
	AA	7 (15.9)	37 (84.1)	
	GA	25 (17.6)	117 (82.4)	0.78
	GG	15 (14.3)	90 (85.7)	
INFL4 rs368234815 TT/DG, N (%)*				
	DG/DG	4 (11.8)	30 (88.2)	
	TT/DG	21 (14.6)	123 (85.4)	0.61
	TT/TT	22 (17.9)	101 (82.1)	
PNPLA3 rs738409 G > C, N (%)*				
	CC	33 (16.6)	166 (83.4)	
	CG	17 (11.9)	126 (88.1)	0.30
PNPLA3 rs738409 G > C, N (%)*				
	GG	2 (6.9)	22 (93.1)	
	CC	33 (16.6)	166 (83.4)	0.17
	CG or GG	19 (11.1)	153 (88.9)	
PNPLA3 rs738409 G > C, N (%)* (only ≤ 65 years old patients)				
	CC	10 (8.4)	109 (91.6)	0.035
	CG or GG	2 (1.8)	109 (98.2)	
TLL1 rs17047200 A > T, N (%)*				
	AA	39 (13.7)	246 (86.3)	
	AT	9 (12)	66 (88)	0.11
	TT	4 (36.4)	7 (63.6)	
TLL1 rs17047200 A > T, N (%)*				
	TT	4 (36.4)	7 (63.6)	0.053
	AA or AT	48 (13.3)	312 (86.7)	
TLL1 rs17047200 A > T, N (%)* (only ≤ 65 years old patients)				
	TT	2 (33.3)	4 (66.7)	0.029
	AA or AT	9 (4.1)	208 (95.9)	

*Not assessed in all subjects.

These associations were confirmed by the multivariable logistic regression analyses performed on patients aged 65 years or less. After adjustment for sex, an adj-OR of 4.69 (95% CI 1.01–22.04) was observed for GG in PNPLA3 rs738409 and an adj-OR of 9.1 (95% CI 1.45–57.3) for TT in TLL-1 rs17047200 (Table 5).

**TABLE 5:** Multivariable logistic regression models on risk factors associated with severe outcome (death or critical/intensive care hospitalization).

	Odds Ratio	95% CI	*p*-Value
**Model 1 (all patients)**			
TLL1 TT vs. (AA or AT)	3.10	0.73–13.2	0.12
SEX (M vs. F)	3.01	1.49–6.05	0.002
AGE (by year of increase)	1.06	1.04–1.09	<0.0001
**Model 2 (all patients)**			
PNPLA3 rs738409 CC vs. (CG or GG)	1.45	0.75–2.78	0.27
SEX (M vs. F)	3.14	1.57–6.29	0.012
AGE (by year of increase)	1.06	1.04–1.08	<0.0001
**Model 3 (patients aged ≤ 65 years)**			
TLL1 TT vs. (AA or AT)	9.1	1.45–57.3	0.018
SEX (M vs. F)	2.13	0.55–8.3	0.275
**Model 4 (patients aged ≤ 65 years)**			
PNPLA3 rs738409 CC vs. (CG or GG)	4.69	1.01–22.04	0.049
SEX (M vs. F)	2.12	0.54–8.4	0.269

*In silico* analysis showed that there are at least 20 cleavage sites on the Spike protein substrate for the TLL-1 protease activity, confirming that TLL-1 is potentially involved in the Spike protein cleavage (Table 6).

**TABLE 6:** Top 20 cleavage sites predicted with SitePrediction ranked by average score.

Rank	Position	Site	N fragment	C fragment	Frequency score	Similarity max score	Similarity max site	Average score	Specificity
1	319 to 326	RVQP.TESI	36.6 kD	104.6 kD	0.003	37.500	QLQKDENI	0.116	>99%
2	401 to 408	VIRG.DEVR	45.8 kD	95.3 kD	0.002	31.111	FYRADQPR	0.058	>99%
3	634 to 641	RVYS.TGSN	71.6 kD	69.6 kD	0.002	26.190	RSYSDRGE	0.054	>99%
4	219 to 226	GFSA.LEPL	25.5 kD	115.6 kD	0.001	40.000	GEAATSPM	0.028	>99%
5	91 to 98	YFAS.TEKS	10.7 kD	130.5 kD	0.001	24.444	YYRADDAN	0.025	>99%
6	990 to 997	EVQI.DRLI	109.9 kD	31.3 kD	0.000	42.105	QLQKDEVI	0.021	>99%
7	1255 to 1262	KFDE.DDSE	139.5 kD	1.7 kD	0.000	42.500	EFTEDQAA	0.017	>99%
8	685 to 692	RSVA.SQSI	76.9 kD	64.2 kD	0.001	16.667	RSYSDRGE	0.015	>99%
9	493 to 500	QSYG.FQPT	56.2 kD	84.9 kD	0.000	30.000	PYYGDEPM	0.008	>95%
10	254 to 261	SSSG.WTAG	29.3 kD	111.9 kD	0.000	35.000	SYAADTAG	0.005	>95%
11	673 to 680	SYQT.QTNS	75.6 kD	65.6 kD	0.000	32.500	SYAADTAG	0.005	>95%
12	983 to 990	RLDK.VEAE	109.1 kD	32.1 kD	0.000	23.684	QLQKDEVI	0.005	>95%
13	545 to 552	GLTG.TGVL	61.9 kD	79.3 kD	0.000	20.000	GEAATSPM	0.005	>95%
14	937 to 944	SLSS.TASA	104.2 kD	36.9 kD	0.000	12.500	SYAADTAG	0.004	>95%
15	787 to 794	QIYK.TPPI	88.0 kD	53.1 kD	0.000	28.947	QLQKDEVI	0.004	>95%
16	863 to 870	PLLT.DEMI	96.3 kD	44.8 kD	0.000	42.105	QLQKDEVI	0.003	>95%
17	659 to 666	SYEC.DIPI	74.2 kD	66.9 kD	0.000	26.667	FYRADQPR	0.003	>95%
18	839 to 846	DCLG.DIAA	93.7 kD	47.4 kD	0.000	20.000	EFTEDQAA	0.003	>95%
19	416 to 423	GKIA.DYNY	47.4 kD	93.8 kD	0.000	34.043	GMVGDDPY	0.003	>95%
20	248 to 255	YLTP.GDSS	28.8 kD	112.3 kD	0.000	24.444	YYRADDAN	0.003	>95%

*The amino acid sequence of Spike protein from UniProt was used as substrate while 17 known cleavage sites for TLL1 were retrieved from MEROPS. Each row represents a cleavage site within Spike protein together with its position and site.*

## Discussion

We have used, for the first time to our knowledge in the field of SARS-CoV-2, nucleic acid extracts generated from swabs during the diagnostic processing COVID-19 to evaluate the host’s genetic profile. This approach, originally devised for other respiratory viruses, had suggested that the IFN lambda system could be a determinant of the outcome of such infections (Rugwizangoga et al., 2019). Albeit we could not find any significant relation between the IFN lambda system and the outcome of COVID-19, the perspective of using genetic material obtained from swabs could be of major relevance for wide, population-based studies of the genetic background of people infected by SARS-CoV-2. In the unresolved pathogenetic scenery of COVID-19, the individuation of genetic variants associated with more prolonged course or with a severe outcome of infection, would support the development of predictive tools useful to stratify subjects by risk class at presentation. Moreover, the individuation of key genes could contribute to a better understanding of the pathways involved in the pathogenesis, giving the basis for rational therapeutic approaches.

As already widely reported, old age and, to a lesser degree, male sex were major determinants in the prognosis of COVID-19 also in our cohort (Onder et al., 2020). Due to unreliability of data sources, we cannot comment on the role of comorbidities in this group. Most comorbidities in the general Italian population are, however, likely to be prevalent in the last decades of life (Italian National Institute of Statistics, 2020. Available at^5^).

Generally speaking, subjects beyond 65 years of age had a seven-fold risk of developing a severe outcome of COVID-19 than their younger counterpart. Moreover, in older patients the presence of comorbidities could represent a confounding factor in identifying other risk factors, as genetic polymorphisms, with weaker associations.

In this setting, the role of other predisposing factors to disease severity, including host’s genetic, is likely to be offset. By converse, in the younger age group, where demographic variables and comorbidities are less prominent, some of the explored host’s polymorphisms of genes linked to innate inflammatory response (PNPLA3) and proteolytic activities (TLL-1) were significantly associated to the worst outcome of COVID-19.

Although no precise pathogenetic pathway can be defined by these observations, some issues deserve considerations. Viral infections are detected by the host innate immune system using pattern recognition receptors (PRRs) activated by pathogen-associated molecular patterns (PAMPs) leading to interferon (IFN) signaling induction. Type III IFNs or lambda IFNs use a heterodimeric receptor (IFNLR1-IL10R2) mainly expressed on the epithelial cells. As proof of the key role type III IFNs in the regulation of immunity response, single nucleotide polymorphisms in genes IFNLs were strongly associated with outcomes to viral infection (Hemann et al., 2017). It has been reported that the homozygosity for IFNL3 (rs12979860) and IFNL4 (rs368234815) variants, overrepresented in African descent, is associated with a reduction of viral clearance in children affected by acute respiratory infections sustained by Rhinovirus and Coronavirus (Rugwizangoga et al., 2019). Since subjects with rs12979860 CC and rs368234815 TT haplotype were reported to be more effective in clearing RNA viruses, possibly due to an up-regulation of inflammatory pathways, we aimed to assess whether the outcome of SARS-CoV-2 infection is conditioned by these polymorphisms. Our results suggest that the polymorphic status of IFNL3/IFNL4 does not affect the rate of infection, since the genotypes are fully in Hardy-Weinberg equilibrium, nor the likelihood of a severe outcome of COVID-19. Hence the suggestion about using IFNLs as an antiviral in COVID-19 patients or in subjects at high risk of infection, currently in clinical trials with peg-IFN L1, would not be supported (Prokunina-Olsson et al., 2020).

COVID-19 pneumonia is characterized by inflammatory exudation of monocytes and lymphocytes. Lung tissues show an abnormal accumulation of CD4 + helper T lymphocytes and CD163 + M2 macrophages recruited by type II pneumocytes in the alveolar spaces. The immunohistochemical evidence, showing strong positivity and site-specific expression, suggest that M2 macrophages play a key role in COVID-19 pathogenesis (Zeng et al., 2020). Mer tyrosine kinase (MERTK) is a major macrophage receptor involved in the clearance of apoptotic cells expressed principally in the subpopulation of M2 macrophages (Zizzo et al., 2012). The polymorphic status of the MERTK gene is able to influence its expression, conditioning M2 polarization of resident macrophages. Genotyping of our patients show that the polymorphic assessment for MERTK does not affect neither the rate of infection, the genotypes being in Hardy-Weinberg equilibrium, nor the likelihood of a severe outcome of COVID-19.

The patatin-like phospholipase domain-containing 3 (PNPLA3) is a triacylglycerol lipase, which mediates triacylglycerol hydrolysis in adipocytes. The PNPLA3 missense variant rs738409 (Ile148Met) (C > G), causing loss of function, is associated with hyperexpression of the NLRP3 inflammasome, leading to increased serum levels of IL-1β and IL18 (Mitsuyoshi et al., 2017). It is known that many viruses and among them SARS-CoV-2, are directly able to induce activation of the NRLP3 inflammasome leading to the cytokines storm probably causing most fatal outcomes (Farag et al., 2020). In our sub cohort of patients ≤ 65 years, the GG PNPLA3 genotype was significantly associated to an increased risk of severe outcome. It is thus conceivable that subjects carrying the GG genotype for rs 738409, having a constitutive upregulation of the NLRP3 inflammasome, develop more severe tissue damage when infected by SARS-CoV-2.

Tolloid Like 1 (TLL-1) is a gene encoding an astacin-like, zinc-dependent, metalloprotease that belongs to the peptidase M12A family. The TLL-1 intronic variant rs17047200 (A > T) was described, for the first time, in 2017 as associated with development of hepatocellular carcinoma after eradication of hepatitis C virus infection (Matsuura et al., 2017). Using *in silico* approach we found that near TLL1 gene maps the Intergenic Long Non-Coding (LINC01179) able to work as an element in *cis* to regulate the gene expression. We evaluate if the polymorphic status relatively to rs17047200 (A > T) of TLL-1 gene is related to the outcome in SARS-Co-V-2 patients. We found that the allelic distribution in our cohort for this SNP is not consistent with Hardey-Weinberg equilibrium being the number of homozygotes TT observed higher (11) respect to the expected (6,3) (Table 3). In addition, the TT genotype in patients aged 65 years or less was found to be significantly associated to an increased risk of severe outcome (*p* = 0.029). This data suggests that the homozygosity TT could be related not only with the occurrence of severe outcome but also with a higher rate of infection. The possible explanation is related to the TLL-1 catalytic domain which shows a relative promiscuity, and many TLL-1 substrates are known. The spike protein (S), a trimeric transmembrane protein of the SARS-CoV-2, after cleavage of the ectodomain, is essential for viral binding and entry into the host cells using the receptor angiotensin converting enzyme 2 (ACE2). The cleavage of S into subunits represents the fundamental step for viral entry in uninfected cells, and SARS-CoV-2 has developed several strategies for proteolytic activation using a large number of host proteases. Among them, furin, trypsin, *trans-*membrane protease/serine (TMPRSS) which cleave S in Golgi apparatus or during virus endosomal uptake, and cathepsins which cleave S during virus entry (Sun et al., 2020). A role of the interaction between complement defense collagens C1q and mannose-binding lectin with TLL-1 in triggering the activation of complement during inflammation and tissue repair has also been described (Lacroix et al., 2017). Among our younger patients, homozygosity for the TT genotype was significantly associated to an increased risk of severe outcome, at a lesser strength than PNPLA3 and further study are needed to understand the mechanism at the bases of this evidence.

Albeit these data suggest that SNPs for PNPLA3 and for TLL-1 may modulate the course of SARSCoV-2 infection, we must acknowledge some limitations of our study. The enrolment cohort was limited in sample size, thus possibly preventing some of the uncommon SNPs to reach significance due to the small number of patients with each variant. Along the same line, the relatively low number of unfavorable outcomes may have curtailed the significance of some allelic variants. The modality of accrual of the cohort through a referral laboratory may have originated some selection and information retrieval biases, and an external validation group has not been tested. Last but not least, a whole genome sequencing approach, rather than a spot evaluation of some individual parameters, would have originated more information. The latter approach, although desirable, was made impossible by the lockdown phase at the time when the study was performed at our Institution.

Patients in our cohort were collected during the early phase of local spreading of the infection, at a time when pre-existing immunity in the general population can be estimated to be non-existent. In this group, the distribution of the low frequency GG genotype distribution of PNPLA3 rs738409 was in accord to the Hardy-Weinberg equilibrium. When assessing whether the polymorphic status of rs17047200 (A > T) of the TLL-1 gene is related to the outcome of COVID-19 patients, we found that the allelic distribution in our cohort for this SNP was not entirely consistent with the Hardy-Weinberg equilibrium, suggesting a possible predisposing role to SARS-CoV-2 infection. Over the last weeks of the first wave, there has been a diffuse, albeit yet unsubstantiated, feeling that the clinical expression of COVID-19 has become less aggressive, and that new cases occurring in areas where SARS-CoV-2 was still actively spreading were less severe. It is unclear whether this was due to an attenuation of viral pathogenicity, to environmental factors or to population characteristics. Whilst the reported low number of deaths due to COVID-19 in our region cannot cause a reduction in the number of subjects with the “unfavorable” genotype, a further unfavorable evolution of the pandemics would possibly cause a reduction in the number of subjects carrying it (Ledda et al., 2021).

In conclusion, we feel that polymorphisms of the host’s genetic determinants, and especially those related to the innate inflammatory response to SARS-CoV-2, should be carefully assessed by a wide-ranging approach, aiming to develop gene profiling tools in order to support an early prediction at the individual level in the course of COVID-19. If host’s polymorphisms are confirmed as determinants of severity, new strategies for identifying vulnerable populations or patients at higher risk for severe disease could be implemented and promoted, improving diagnosis, treatment and prognosis of COVID-19.

## Data Availability Statement

The data presented in the study are deposited in the dbSNP repository: accession number NM 025225.2 for PNPLA3; accession number NM 001204760.1; NM_012464.4 for TLL1; accession number NM_006343.2 for MERTK; accession number NR_074079.1 for IFNL4; accession number NM_001276254.2; NR_074079.1 for IFNL3.

## Ethics Statement

The studies involving human participants were reviewed and approved by the University Hospital “P. Giaccone” of Palermo. The patients/participants provided their written informed consent to participate in this study.

## Author Contributions

SG, EA, FV, ACa, and ACr: conceptualization, writing, review, and editing. SG, EA, and RP: methodology. DS: software. SG, EA, DS, and ACa: validation and formal analysis. SG, EA, RP, CM, SP, TP, FT, and DS: investigation, resources, and data curation. ACa and ACr: supervision. All authors have read and agreed to the published version of the manuscript.

## Conflict of Interest

The authors declare that the research was conducted in the absence of any commercial or financial relationships that could be construed as a potential conflict of interest.

## Footnotes

1 https://covid19.who.int/

2 https://www.covid19hg.org/

3 https://www.cdc.gov/coronavirus/2019-ncov/lab/rt-pcr-panel-primer-probes.html

4 https://www.r-project.org/

5 https://www.istat.it/it/files/2017/09/Condizioni_Salute_anziani_anno_2015.pdf

## References

[B1] Centers for Disease Control and Prevention (2020). *Research Use Only 2019-Novel Coronavirus (2019-nCoV) Real-time RT-PCR Primers and Probes.* Atlanta: Centers for Disease Control and Prevention.

[B2] Chan J. F. Yuan S. Kok K. H. (2020). A familial cluster of pneumonia associated with the 2019 novel coronavirus indicating person-to-person transmission: a study of a family cluster. *Lancet* 395 514–523. 10.1016/S0140-6736(20)30154-9 31986261 PMC7159286

[B3] COVID-19 Host Genetics Initiative (2020). The COVID-19 Host Genetics Initiative, a global initiative to elucidate the role of host genetic factors in susceptibility and severity of the SARS-CoV-2 virus pandemic. *Eur. J. Hum. Genet.* 28 715–718. 10.1038/s41431-020-0636-6 32404885 PMC7220587

[B4] De la Rica R. Borges M. Gonzalez-Freire M. (2020). COVID-19: In the Eye of the Cytokine Storm. *Front. Immunol.* 11:558898. 10.3389/fimmu.2020.558898 33072097 PMC7541915

[B5] Farag N. S. Breitinger U. Breitinger H. G. El Azizi M. A. (2020). Viroporins and inflammasomes: a key to understand virus-induced inflammation. *Int. J. Biochem. Cell Biol.* 122:105738. 10.1016/j.biocel.2020.105738 32156572 PMC7102644

[B6] Hemann E. A. Gale M. Jr. Savan R. (2017). Interferon Lambda Genetics and Biology in Regulation of Viral Control. *Front. Immunol.* 8:1707. 10.3389/fimmu.2017.01707 29270173 PMC5723907

[B7] Italian National Institute of Statistics (2020). *ANZIANI: LE CONDIZIONI DI SALUTE IN ITALIA E NELL’UNIONE EUROPEA.* Rome: Italian National Institute of Statistics.

[B8] Joynt G. M. Wu W. K. (2020). Understanding COVID-19: what does viral RNA load really mean? *Lancet Infect. Dis.* 20 635–636. 10.1016/s1473-3099(20)30237-1 32224308 PMC7118539

[B9] Lacroix M. Tessier A. Dumestre-Pérard C. (2017). Interaction of Complement Defence Collagens C1q and Mannose-Binding Lectin with BMP-1/Tolloid-like Proteinases. *Sci. Rep.* 7:16958. 10.1038/s41598-017-17318-w 29209066 PMC5717261

[B10] Ledda C. Carrasi F. Longombardo M. T. Paravizzini G. Rapisarda V. S. A. R. S. - (2021). CoV-2 Seroprevalence Post-First Wave among Primary Care Physicians in Catania (Italy). *Trop. Med. Infect. Dis.* 6:21. 10.3390/tropicalmed6010021 33572221 PMC7930996

[B11] Li X. Wang W. Zhao X. Zai J. Zhao Q. Li Y. (2020). Transmission dynamics and evolutionary history of 2019-nCoV. *J. Med. Virol.* 92 501–511. 10.1002/jmv.25701 32027035 PMC7166881

[B12] Matsuura K. Sawai H. Ikeo K. Ogawa S. (2017). Genome-Wide Association Study Identifies TLL1 Variant Associated With Development of Hepatocellular Carcinoma After Eradication of Hepatitis C Virus Infection. *Gastroenterology* 152 1383–1394. 10.1053/j.gastro.2017.01.041 28163062

[B13] Mitsuyoshi H. Yasui K. Hara T. (2017). Hepatic Nucleotide Binding Oligomerization DomainLike Receptors Pyrin Domain-Containing 3 Inflammasomes Are Associated With the Histologic Severity of Non-Alcoholic Fatty Liver Disease. *Hepatol. Res.* 47:14591468. 10.1111/hepr.12883 28245087

[B14] Onder G. Rezza G. Brusaferro S. (2020). Case-Fatality Rate and Characteristics of Patients Dying in Relation to COVID-19 in Italy. *JAMA* 323 1775–1776. 10.1001/jama.2020.4683 32203977

[B15] Phan L. Jin Y. Zhang H. (2020). *ALFA: Allele Frequency Aggregator.* Maryland: National Center for Biotechnology Information.

[B16] Prokunina-Olsson L. Alphonse N. Dickenson R. E. (2020). COVID-19 and emerging viral infections: The case for interferon lambda. *J. Exp. Med.* 217:e20200653. 10.1084/jem.20200653 32289152 PMC7155807

[B17] R Core Team (2019). *R Software version 3.6.1 (2019-07-05).* Vienna: R Core Team.

[B18] Rawlings N. D. Barrett A. J. Thomas P. D. Huang X. Bateman A. Finn R. D. (2018). The MEROPS database of proteolytic enzymes, their substrates and inhibitors in 2017 and a comparison with peptidases in the PANTHER database. *Nucleic Acids Res.* 46 D624–D632. 10.1093/nar/gkx1134 29145643 PMC5753285

[B19] Rugwizangoga B. Andersson M. E. Kabayiza J. C. (2019). IFNL4 Genotypes Predict Clearance of RNA Viruses in Rwandan Children With Upper Respiratory Tract Infections. *Front. Cell Infect. Microbiol.* 9:340. 10.3389/fcimb.2019.00340 31637221 PMC6787560

[B20] Sinha P. Matthay M. A. Calfee C. S. (2020). Is a “Cytokine Storm” Relevant to COVID-19? *JAMA Intern. Med.* 180 1152–1154. 10.1001/jamainternmed.2020.3313 32602883

[B21] Sun J. He W. T. Wang L. (2020). COVID-19: Epidemiology, Evolution, and Cross-Disciplinary Perspectives. *Trends Mol. Med.* 26 483–495. 10.1016/j.molmed.2020.02.008 32359479 PMC7118693

[B22] Tam V. Patel N. Turcotte M. Bossé Y. Paré G. Meyre D. (2019). Benefits and limitations of genome-wide association studies. *Nat. Rev. Genet.* 20 467–484. 10.1038/s41576-019-0127-1 31068683

[B23] Verspurten J. Gevaert K. Declercq W. Vandenabeele P. (2009). SitePredicting the cleavage of proteinase substrates. *Trends Biochem. Sci.* 34 319–323. 10.1016/j.tibs.2009.04.001 19546006

[B24] World Health Organization (2020). *World Health Organization coronavirus disease situation dashboard.* Geneva: World Health Organization.

[B25] Zeng Z. Xu L. Xie X. Y. (2020). Pulmonary Pathology of Early Phase COVID-19 Pneumonia in a Patient with a Benign Lung Lesion. *Histopathology* 2020:14138. 10.1111/his.14138 32374419 PMC7267508

[B26] Zizzo G. Hilliard B. A. Monestier M. Cohen P. L. (2012). Efficient clearance of early apoptotic cells by human macrophages requires M2c polarization and MerTK induction. *J. Immunol.* 189 3508–3520. 10.4049/jimmunol.1200662 22942426 PMC3465703

